# Case report: Two cases of Poirier-Bienvenu neurodevelopmental syndrome and review of literature

**DOI:** 10.3389/fped.2023.967701

**Published:** 2023-03-20

**Authors:** Xiaolan Chen, Yunli Han, Xing Li, Shiqin Huang, Hai Yuan, Yuanhan Qin

**Affiliations:** Department of Pediatrics, The First Affiliated Hospital of Guangxi Medical University, Nanning, China

**Keywords:** Poirier-Bienvenu neurodevelopmental syndrome (POBINDS), CSNK2B gene, variant, seizure, MOSAIC, developmental delay

## Abstract

The Poirier-Bienvenu neurodevelopmental syndrome (POBINDS) is a rare disease caused by mutations in the CSNK2B gene, which is characterized by intellectual disability and early-onset epilepsy. Mosaicism has not been previously reported in CSNK2B gene. POBINDS is autosomal dominant and almost all reported cases were *de novo* variants. Here, we report two patients were diagnosed with POBINDS. Using Whole Exome Sequencing (WES), we detected two novel CSNK2B variants in the two unrelated individuals: c.634_635del (p.Lys212AspfsTer33) and c.142C > T (p.Gln48Ter) respectively. Both of them showed mild developmental delay with early-onset and clustered seizures. The patient with c.634_635del(p.Lys212AspfsTer33) variant was mutant mosaicism, and the proportion of alleles in peripheral blood DNA was 28%. Further, the literature of patients with a *de novo* mutation of the CSNK2B gene was reviewed, particularly seizure semiology and genotype-phenotype correlations.

## 1. Introduction

The protein kinase CK2 (CK2) is a small family of eosinophilic serine/threonine kinases that phosphorylate various substrates involved in biological processes such as apoptosis, cell proliferation, and DNA damage responses (1). CK2 consists of four subunits, including catalytic subunit α, α′ and two β regulatory subunits (2). CK2β, which is encoded by the CSNK2B (OMIM# 115441) gene located on human chromosome 6p21.33, is a prerequisite for the formation and normal function of CK2 (3, 4). CSNK2B gene is broadly expressed in various tissues. Li et al. (5) noted that CSNK2B gene is highly expressed in the developing prefrontal cortex and less expressed in childhood and adulthood. It has been confirmed that CK2β has a negative effect on cell proliferation in general, but the reasons for the different effects of CK2β in different cell lines remain unclear (6).

In 2017, two splice site mutations in CSNK2B gene were first reported to cause intellectual disability with or without myoclonic epilepsy (7). At present, the neurodevelopmental disorder caused by the pathogenic mutation of CSNK2B gene is called Poirier-Bienvenu neurodevelopmental syndrome, also known as CSNK2B- related disorder or CSNK2B encephalopathy (8). Previous studies have suggested that the syndrome is a neurological disorder characterized by early onset epilepsy and developmental disorders, and its phenotype is highly heterogeneous (5, 9, 10). To date, 65 patients with *de novo* CSNK2B mutations have been reported in the literature. Mosaicism has not been previously reported in CSNK2B gene. There is still a lack of systematic investigation of seizure semiology and genotype-phenotype correlations in patients with CSNK2B variants.

Here, we report two patients with CSNK2B gene variants presented with seizure, one of which was mutant mosaicism. Furthermore, we evaluated the clinical phenotype and genetic findings of two previously unreported cases of CSNK2B mutations and all patients previously described with this mutation to assess genotype -phenotype correlations.

## 2. Materials and methods

### 2.1. Subjects

Two subjects from two unrelated Chinese families with epilepsy of unknown etiology were analyzed. Two patient-parent trios were clinically evaluated and underwent Trio-WES accompanied by CNV and Sanger sequencing. After whole exon sequencing, patient 1 was also subjected to ultra-deep sequencing (10,000×).

### 2.2. CSNK2B sequence and bioinformatic analysis

We describe the sequencing of genomic DNA from peripheral blood and the bioinformatic analysis of the CSNK2B variants. Pathogenicity of novel variants was determined based on the standard guidelines of the American College of Medical Genetics and Genomics (ACMG) (11).

### 2.3. Systematic literature review

We conducted a systematic literature review focused on Poirier-Bienvenu neurodevelopmental syndrome by searching the PubMed database, CNKI and Wanfang Data using the keywords “CSNK2B” (until May 2022). Clinical studies with CSNK2B mutations were reviewed for all publications. All mutations identified met ACMG-Association for Molecular Pathology classification criteria as “pathogenic” or “likely pathogenic”.

## 3. Results

### 3.1. Clinical features of two patients

CSNK2B variants were found in two patients who shared mild developmental delays and myoclonic seizures without dysmorphic features, but exhibited different features.

Patient 1 was born at term following an uneventful pregnancy and delivery. Weight, height, and head circumference measurements were normal at birth. He developed febrile convulsions at the age of 1 year 11 months, twice a year. Seizures started at the age of 3 years 8 months and language development was delayed at this age. Generalized tonic-clonic seizures were present in clusters of 3–5 seizures per day at the beginning. One month after onset, the boy began to have focal seizures (2–3 per day) and myoclonic seizures (hundreds per day). Seizures increased significantly with fever. We evaluated the Gesell Developmental Schedules Score (gross motor DQ: 88, fine motor DQ: 88, adaptive behavior DQ: 90, language DQ: 79, personal social DQ: 85) at the age of 3 years 9 months. The electroencephalogram (EEG) was normal at 1 year 11 months of age and turned to be abnormal at 3 years 9 months of age with slow background, diffuse spike-slow, multiple spike-slow, and sharp wave discharges. Brain computed tomography (CT) was normal at 1 year 11 months of age, and brain magnetic resonance imaging (MRI) had slightly widened transparent without diaphragmdelayed myelination and cerebellar hypoplasia at 3 years 9 months of age. The metabolic analysis and chromosomal karyotype analysis were normal. After treatment with levetiracetam (LEV), oxazepane (OXC), topiramate (TPM) and valproic acid (VPA), the seizures failed to be controlled. Lamotrigine (LTG) was then used in combination to show significant efficacy. The EEG returned to normal at 4 years and 3 months of age. The seizures were controlled from 4 years old until the present time with only VPA (20 mg/kg/day) and LTG (3.5 mg/kg/day).

Patient 2 was a girl with epilepsy and mild intellectual and developmental delays. She started walking at 15 months and speaking only several words at 3 years and 4 months of age, and we evaluated the Gesell Developmental Schedules Score (gross motor DQ: 81, fine motor DQ: 66, adaptive behavior DQ: 56, language DQ: 56, personal social DQ: 56) at this age. The patient had her first myoclonic seizure at 1 year 5 months with a frequency of 5–10 times per day. Due to the limited medical care in local hospital, she was not diagnosed with epilepsy until 3 years and 4 months old. The EEG at 3 and 4 months of age were abnormal with slow background, multifocal spikes, diffuse spike-slow, multiple spike-slow, and sharp wave discharges. Brain MRI showed slight myelination delays. Metabolic and chromosomal karyotype tests were all negative. The seizures were controlled with VPA (20 mg/kg/day) and the EEG was normal at age 3 years and 10 months.

### 3.2. Identifcation of CSNK2B variants

**Figure 1:**
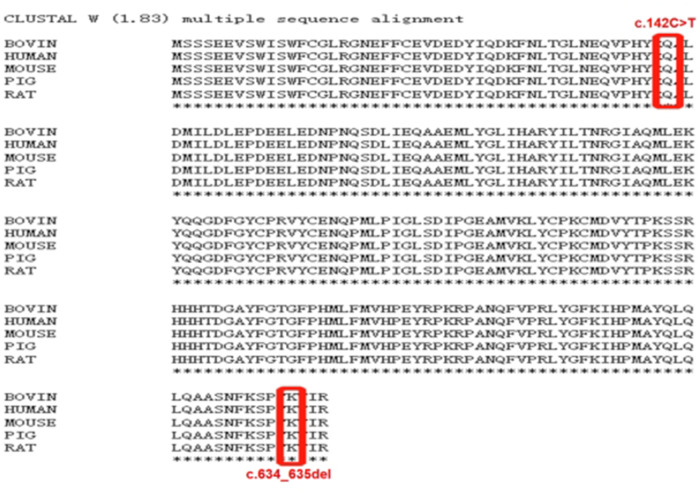
Clustal W(1.83) multiple sequence alignment. The CSNK2B variants marked in red is highly conserved between species.

De novo CSNK2B variants were detected in two probands and confirmed by Sanger sequencing. Two patients had no pathogenic variation found in other epilepsy or intellectual disability (ID)/developmental delay (DD) candidate genes and CNVs. The mosaic variant of c.634_635del (p.Lys212AspfsTer33) was identified in patient 1, the allelic fraction in the blood was 28%. The variant of c.142C > T (p.Gln48Ter) was identified in patient 2. Two *de novo* variants have not been found in the gnomAD, ClinVar database and HGMD database. Neither parent has a similar disease or genetic mutation.Two amino acid residues for variant sites were highly conserved across species (Figure 1). The pathogenicity of c.634_635del (p.Lys212Asp fsTer33) mosaic variant was classified as likely pathogenic and c.142C > T (p.Gln48Ter) variant was classified as likely pathogenic in accordance with the ACMG guideline (Table 1).

**Table 1:** Clinical interpretation of variants detected in CSNK2B by ACMG guideline.

Patient	Variant (NM_001320)	Evidence for pathogenicity based on ACMG guideline				Category
		Very strong	strong	Moderate	Supporting	
1	c.634_635del (p.Lys212Asp fsTer33)	/	PS2	PM2	/	LP
2	c.142C > T (p.Gln48Ter)	PVS1	PS2	PM2	/	P

PVS, pathogenic very strong; PS, pathogenic strong; PM, pathogenic moderate; PP, pathogenic supporting; P, pathogenic; LP, likely pathogenic.

### 3.3. Phenotype of patients with CSNK2B variants

65 individuals with POBINDS have been reported to date (5, 7–10, 12–20). Therefore, with our two patients, 67 patients with POBINDS are the subjects of this study. The main clinical, genetic, EEG, and MRI features are summarized.

The overall patient population includes 43 males and 24 females, aged between 5 months and 36 years at the time of reporting. Epilepsy was diagnosed in 57/67 patients (85.07%). With a range of 3 days to 7 years, the median age at epilepsy onset was 6 months. The seizures onset occurred in the neonatal period in 4 patients, within 6 months after birth in 54.39%, within the first year of life in 73.68%, and within 3 years of age in 96.49%. There were 56 patients with seizure types available for review. Of those, 38 (66.67%) had only one type of seizure, and 19 (33.33%) individuals had multiple types, including generalized tonic-clonic seizure (GTCS), myoclonic, myoclonic-absence, myotonic-atonic, absence/atypical absence, infantile spasm (IS), tonic, atonic, tonic-spasms, drop attacks, focal, and secondary generalized seizures. The most common type of epilepsy was GTCS, which was occurred in 34 (59.65%) patients.

In addition to the different phenotypes of epilepsy, seizure outcome was varied. Our study failed to obtain medication information on one patient, and another patient did not receive any antiseizure medications (ASMs). 55 patients received antiseizure therapy. 54.54% (30/55) were controlled on monotherapy or two drugs. Twenty-five of 55 (45.45%) patients were medically refractory or required multiple medications. Four patients were treated with ketogenic diet (KD) in combination with other ASMs, which showed no effects on seizures. One patient with five seizure types failed to control the seizures after trying 12 drugs, including ACTH. A patient with myoclonic epilepsy underwent VNS implantation after multi-drug treatment and the seizures remained uncontrolled. There have been no cases of SUDEP. Except for 3 patients with no medication information or unknown seizure type, 12 patients (33.33%, 11/36) with only one seizure type and 12 patients (66.67%, 12/18) with two or more types required multi-drug therapy or were drug-resistant (*p* = 0.02, Pearson Chi-square test). Additionally, seizures were well controlled in 85.00% (17/20) of patients with only GTCS when treated with monotherapy. Four of five patients with only focal seizure were controlled on monotherapy or two drugs. Three of the five patients with only myoclonic seizure were drug resistant or required multi-drug therapy. One patient had no myoclonic-absence seizure after medication, but another one who had only myoclonic-atonic seizure was drug-resistant. The ASMs most often to be used were VPA (25 cases), LEV (24 cases), TPM (11 cases), LTG (10 cases), and zonisamide (8 cases). To date, no ASMs was reported to exacerbate seizures. There is no established treatment for CSNK2B-related epilepsy.

The cognitive development of all 67 patients with CSNK2B mutations has been monitored. Twenty of 67 (29.85%) patients with cognitive information had severe ID/DD, seven (10.45%) had moderate ID/DD, 34 (50.75%) had mild ID/DD, and six (8.95%) had normal ID/DD. 51 of 57(89.47%) patients with epilepsy presented ID/DD. Moderate-severe ID/DD was observed in 22 of 57 (38.59%) patients with CNSK2B- related epilepsy and in 5 of 10 (50.00%) patients without epilepsy. There was no significant difference on cognition between patients with epilepsy and those without epilepsy (*p* = 0.498, Pearson Chi-square test). 13 of 17 epilepsy patients with severe ID/DD were medically refractory or required multiple medications. Seizures were well controlled in 6 patients with normal development. Patients with severe seizures showed more severe ID/DD.

Neurologic examination showed anomalies in lots of patients. Hypotonia occurred in 20 patients (29.85%, 20/67). Additional neurologic features included dystonia, nystagmus, wide-based gait, and cerebellar symptoms. Autistic features were noted in 12 patients (17.91%, 12/67), including 10 epilepsy individuals (17.54%, 10/57). Additional neuropsychological disorder included aggression, hyperactivity, anxiety disorder, social dysfunction, dyssomnia, and poor coordination. Facial dysmorphisms were noted in 24 individuals (35.82%, 24/67).There was no recognizable facial pattern nor any facial features characteristic of the CSNK2B mutations. Microcephaly was observed in seven individuals (10.45%, 7/67), and macrocephaly was observed in four individuals each (5.97%, 4/67). Other deformities and abnormalities were observed, including heart deformities, unilateral polydactyly, short fingers, tapered distal phalanges of finger, cleft soft palate, laryngeal cleft, undescended testes, scoliosis, renal hypoplasia, asymmetry in limb creases, and uterine agenesis. Failed to thrive or short stature may be another common feature of the disorder. 17 of 67 (25.37%) patients had failure to thrive or short stature. Three patients responded well to growth hormone therapy, including one without growth hormone deficiency but with bone age delay. Two patients were diagnosed as precocious puberty. Some children had other systemic abnormalities, including pectus excavatum, nocturnal enuresis, dysphagia, intermittent ptosis, gastroesophageal reflux, hearing loss, aspiration pneumonias, mild hirsutism, hypermetropia, soft skin, hepatomegaly, anemia, and laryngomalacia.

EEG and MRI were variable with no typical feature. EEG data was available for review in 42 patients; 33 individuals (78.57%, 33/42) displayed epileptiform discharges. The findings of EEG abnormalities was wide, and no specific EEG abnormalities were found. Generalized and multifocal spike/polyspikes/spike-slow waves were the most common patterns observed on EEG, with or without slow background activity. Neuroimaging was available for 46 individuals and showed abnormalities in 16 (34.78%, 16/46) patients. Delayed myelination was mild to morderate in three individuals. Two patients had hippocampus abnormalities. Abnormal signal in the cerebral white matter was observed in two cases. Two patients showed low volume pons, and another one show T2 hyperintensity and restricted diffusion of pontine central tegmental tracts. Cerebral atrophy was noted in one case. Chiari type I malformation and syringomyelia was observed in one case. Other findings include periventricular gliosis, germinolytic cysts, mega cisterna magna, enlargement of the cerebrospinal fluid spaces, and gyral simplification.

**Figure 2:**
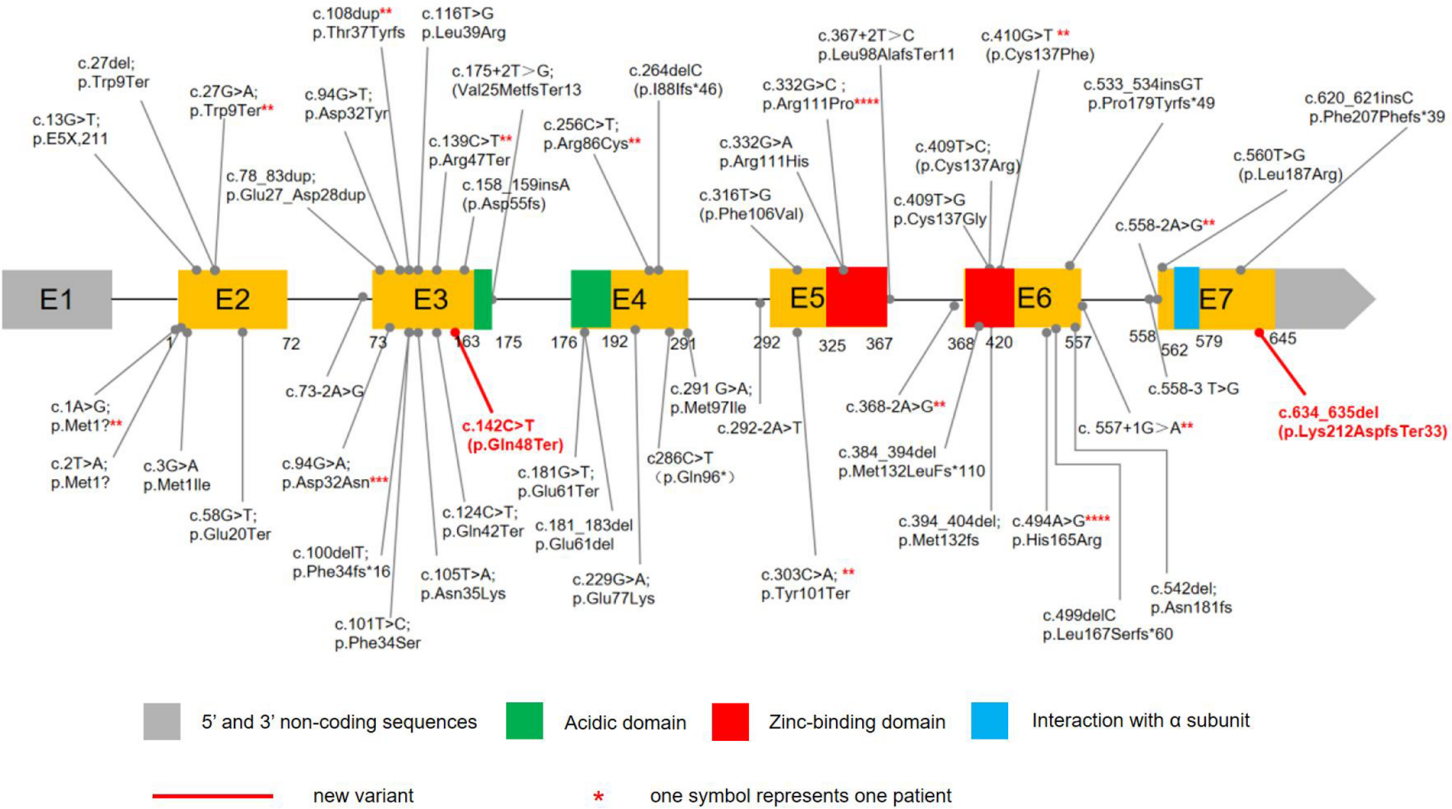
The variants in CSNK2B DNA sequence. 65 children with POBINDS caused by CSNK2B variants have been reported, with various mutation types, including missense, nonsense, splice site, start loss, frameshift, and in-frame duplication mutations. Our cases had two de novel variants. c.634_635del was a mosaic variant, c.142C > T was a nonsense mutation.

CSNK2B contains 7 exons, and mutations have been found in Exons 3 (25.37%,17/67), 6(25.37%,17/67), 2(13.43%,9/67), 4(11.94%,8/67), 5(14.93%,10/67), and 6(8.96%,6/67), whereas no mutation was reported for Exons 1. A total of 50 distinct CSNK2B mutations have been identified in 67 patients (Figure 2). Most variants (94.03%, 63/67) were *de novo*, including in one patient who was mosaic. Missense variants are the most common types of variants (40.30%, 27/67). Other variants included 12 nonsense, 12 frameshift, 11 splice site, 3 start-loss, one mosaic variant, and one in-frame duplication. In total, 13 variants were recurrent, including c.332G > C in four, c.494A > G in four, c.94G > A in three patients, and c.2T > A, c.27G > A, c.108dup, 139C > T, c.256C > T, c.303C > A, c.368-2A > G, c.410G > T, c.557+1G > A, c.558-2A > G had 2 cases each.So far, functional studies have been performed on four splice sites, three frameshift, and one missense variants(c.175+2T > G, c.292-2A > T, c.367+2T > C, c.558-3T > G, c.499delC, c.533_534insGT, c.558_709del, c.494A > G). Fourteen patients with mutations located in the zinc-binding domain, including nine loci of mutations. 12 patients in the zinc-binding domain had seizures, of which 10 had only one seizure type, 8 had only GTCS, and only one had epilepsy resistance.

We compared the phenotypes of 27 patients with missense variants and 40 patients with non-missense mutations (Table 2). There was no significant difference in other phenotypes except that non-missense mutations were more likely to have multiple types of seizures than missense mutations. There was no significant correlation between the location of variation and phenotypic severity. Phenotypic heterogeneity exists even with the same variation. The recurrent c.494A > G variant has been described in four unrelated patients with severe ID/DD and seizures. The onset of seizure in all individuals was in the neonatal period. Two patients had myoclonic seizures, and two patients had focal seizures. Drug-resistant epilepsy was present in three patients, the other one had seizures controlled on monotherapy (LEV). Recurrent c.332G > C variant was also noted in 4 patients. Three patients were diagnosed with epilepsy, of which two had GTCS with mild ID/DD and one had myoclonic seizure with moderate ID/DD. One patient with GTCS required 3 antiepileptic drugs for seizure control, the other two patients had seizures controlled on monotherapy. Two patients with c.303C > A variant were identical twin boys with severe ID/ DD and seizures. The onset age of epilepsy was infancy, the seizure type was myoclonic seizure and atonia seizure, all were drug refractory epilepsy, accompanied by dystonia, abnormal gait, microcephaly, and facial deformity.

**Table 2:** Comparison of effects of variant type on clinical manifestations.

Phenotypes	Missense variants	Non-missense variants	*p*-value (chi-square)
Moderate to severe ID/DD	13/27 (48.15%)	14/40 (35.00%)	0.282
Seizure	20/27 (74.07%)	37/40 (92.50%)	0.084
**Age at seizure onset**
<12 months	16/20 (80.00%)	26/37 (70.27%)	0.631
≥12 months	4/20 (20.00%)	11/37 (29.73%)	
GTCS	10/20 (50.00%)	24/37 (64.86%)	0.275
Multiple seizure types	5/20 (25.00%)	22/37 (59.46%)	0.013
Drug-resistant/multiple medications	8/20 (40.00%)	17/37 (45.95%)	0.666

## 4. Discussion

Variants of CSNK2B have been reported in patients with ID/DD, epilepsy, short stature, dysmorphic features, and behavioral disorders. CSNK2B variants have been reported in 65 cases, and 55 of them had epilepsy. Here, we found two novel CSNK2B variants and reported the first patient with mosaic variant to our knowledge. Two patients displayed DD and Epilepsy. Bioinformatics analysis and functional study supported the deleterious effect in 142C > T variant, and c.634_635del was identified in a likely pathogenic variant. Two variants segregated in the family, and WES detected no other likely pathogenic variants. Combining our cases with previously published cases, we found that 85.07% of the subjects had epilepsy. In view of the high prevalence of seizures in this population, all individuals with CSNK2B variants should undergo an epilepsy evaluation. Our results were similar to those in previous literature, suggesting that phenotypic heterogeneity caused by CSNK2B gene variants can range from drug-sensitive GTCS epilepsy to refractory epilepsy, with varying degrees of ID/DD (9, 10, 19). The seizure onset was within 6 months after birth in more than half of patients, with the first year of life in 73.68% of individuals, and almost all patients had seizures before the age of three, indicating an infantile onset and age-dependent epilepsy. 91.05% patients of POBINDS presented ID/DD, ID/DD was co-present in 89.47% of epilepsy patients, which was more severe in patients with more severe seizure phenotypes. It appears that ID/DD is a common comorbidity of CSNK2B-related epilepsy and that severe epilepsy may adversely affect cognitive performance. But there might be no difference in cognitive outcomes between individuals with CSNK2B- related epilepsy and those without epilepsy. This may be related to the fact that the last follow-up time of some patients was infancy and there were few cases, and observation of more cases and long-term follow-up are needed to further assess cognitive function. At present, the reported data of cognitive impairment after onset of epilepsy was incomplete, and it is still not possible to judge whether epilepsy will aggravate cognitive impairment.

We found that epilepsy outcomes among patients with CSNK2B variants are highly variable. One patient did not receive any treatment, and one patient had no treatment information; 54.54% were controlled on monotherapy or two drugs. Thus far, none of the patients with CSNK2B-related epilepsy has died from seizures, suggesting a low risk of death due to CSNK2B-related epilepsy. Seizure semiology in CSNK2B-related epilepsy was variable, and 14 seizure types have been reported. At the time of reporting, it is impossible to capture the full spectrum of seizure types. Other types of seizures may occur at follow-up. Our data suggest that patients with multiple seizure types are more resistant to drugs than those with a single seizure type, and epilepsy with GTCS alone is most easily controlled. Based on the limited data available, it appears that CSNK2B-related epilepsy is not easy to treat, but has responded relatively well to VPA. And the preferred antiepileptic treatment with VPA may have a better prognosis. Mosaic variant patient responded well to LTG after failing VPA and other medications, and LEV, TPM alone can control seizures in some patients. To date, the mechanism of epilepsy caused by CSNK2B mutation is not clear, and the above-mentioned drugs have different antiepileptic mechanisms, the target of antiepileptic treatment cannot be further defined. Although seizures were controlled in more than half of patients, larger prospective studies are necessary to identify more effective therapies.

We note that neuropsychiatric abnormalities were described in some patients, particularly autism. Our data showed that the prevalence of CSNK2B-associated epilepsy comorbid autism is lower than that of Dravet syndrome (33.6%) (21). Hypotonia was the most prominent features of neurological disturbance. Okur-chung syndrome (OCNS), which is caused by a CSNK2A1 gene variant, had a higher rate of dystonia, occurring in more than two-thirds of patients (22). Previous studies have shown that CSNK2B gene knockout in mice can lead to muscle weakness by affecting mitochondrial function and neuromuscular junction (23, 24), but muscle weakness phenotype has not been described in all clinical cases at present. This suggests that we need to pay attention to distinguish between muscle weakness and hypotonia. The facial dysmorphisms found in more than one-third of individuals did not provide any facial features suggestive for CSNK2B variants. Microcephaly and macrocephaly were observed in some patients, and one in four patients had failure to thrive (FTT) or short stature. FTT and short stature are also common in OCNS, often with growth hormone deficiency or pituitary abnormalities (25). The cause of growth hormone deficiency or pituitary abnormalities in POBINDS and OCNS has not been clarified. LHX3 can serve as a substrate for CK2 (26). Previous studies believed that it may be caused by CK2's failure to phosphorylate LHX3 protein normally (25). LHX3 is essential for pituitary development in man, which is required for differentiation of four types of anterior pituitary cell and for establishing a normal cohort of corticotropes by birth (27). CK2 act directly on the GHR at the cell surface and counteract GHR signaling (28). It is recommended that the head circumference, body growth and nutritional status be monitored regularly and that growth hormone be tested as necessary. Three patients underwent growth hormone therapy and showed good responses (16, 19), suggesting that growth hormone supplementation is an effective treatment. Some patients have neuroimaging abnormalities without specific manifestations, which may occur at different sites.

Although all genes except Exon 1 of CNK2B gene have mutations, Exon 3 and 6 have more mutations, highlighting the importance of these regions. We found 13 recurrent variants, c.332G > C, c.494A > G, c.94G > A are possible mutational “hot spots” in CSNK2B. Only a few CSNK2B variants are located in functional domains. Li et al. (5) believed that the zinc-binding domain might be the hotspot of CSNK2B-related epilepsy, and we also found that most patients in the zinc domain had only GTCS and were easily controlled. The same variant site can cause different degrees of phenotype, suggesting that there may be no obvious correlation between the position of variation and the severity of phenotype. For CSNK2B, it is notable that the epileptic phenotype caused by non-missense mutations may be more severe than missense mutations. However, due to the small number of cases, the phenotypic characteristics of different variants still remain not thoroughly explicit. POBINDS displays a phenotypic heterogeneity, suggesting there is no clear genotype-phenotype relationship.

## 5. Conclusion

In conclusion, our study expands the number of patients with POBINDS that has been reported and describes two new variants, of which the CSNK2B gene mosaic variant is reported for the first time. It provides an overview of all POBINDS cases, especially patients with CSNK2B-related epilepsy. The phenotypic spectrum of POBINDS includes ID/DD, early-onset epilepsy, congenital anomalies, FTT or short stature. Individuals with multiple seizure types showed more severe phenotype and were suffering more to contain the symptoms of seizure, whereas patients with single seizure type were easier to control. The phenotype of recurrent variants was highly heterogeneous and there was no obvious correlation between phenotype and mutation location. There is currently no targeted treatment for this disease. The pathogenesis of epilepsy caused by CSNK2B gene variation is still unclear, and further investigation of clinical data of more patients and elucidation of the pathogenesis are helpful for precise treatment.

## Data Availability

The datasets for this article are not publicly available due to concerns regarding participant/patient anonymity. Requests to access the datasets should be directed to the corresponding author.
