# Supplementary material for: Case report: Two cases of Poirier-Bienvenu neurodevelopmental syndrome and review of literature
Source: Front Pediatr. 2023 Mar 20;11:967701. doi: 10.3389/fped.2023.967701 (PMC10067874; doi:10.3389/fped.2023.967701)
Supplement: Supplementary file 1 [file Datasheet1.doc]

**Supplementary Information**

**Table S1-1 The Genotype and Clinical Phenotype of Patients with *CSNK2B* Mutations**

| PATIENT NUMBER | CSNK2B MUTATION | MUTATION TYPE | GENDER | ID/DD | AUTISTIC TRAITS | FTT/Short Stature | Cloverleaf skull | Facial dysmorphy | Hypotonia/Hypertonia/Dystonia |
| --- | --- | --- | --- | --- | --- | --- | --- | --- | --- |
| 1 | c.367+2T＞C | splice site | MALE | SEVERE | YES |  | NO | YES | hypotonia |
| 2 | c.175+2T＞G | splice site | MALE | SEVERE |  |  | NO | YES |  |
| 3 | c.108dup | frameshift | MALE | MILD |  |  | NO | NO |  |
| 4 | c.533_534insGT | frameshift | FEMALE | SEVERE |  | Short Stature,  Precocious Puberty | NO | NO |  |
| 5 | c.494A>G | missense | MALE | SEVERE |  |  | NO | YES | dystonia |
| 6 | c.560T>G | missense | MALE | SEVERE |  |  | NO | NO |  |
| 7 | c.620_621insC | frameshift | FEMALE | MILD |  |  | NO | NO |  |
| 8 | c.13G>T,c.256C>T | nonsense | FEMALE | MODERATE |  |  | NO | NO |  |
| 9 | c.409T>G | missense | FEMALE | NO |  |  | NO | NO |  |
| 10 | c.264delC | frameshift | MALE | NO |  |  | NO | NO |  |
| 11 | c.410G>T | missense | FEMALE | NO |  |  | NO | NO |  |
| 12 | c.332G>C | missense | MALE | MILD |  |  | NO | NO |  |
| 13 | c.332G>C | missense | FEMALE | MILD |  |  | NO | NO |  |
| 14 | c.368-2A>G | splice site | FEMALE | MILD |  |  | NO | NO |  |
| 15 | c.139C>T | nonsense | MALE | MILD |  |  | NO | YES | Hypotonia |
| 16 | c.557+1 | splice site | MALE | MILD |  |  | NO | NA |  |
| 17 | c.410G>T | missense | FEMALE | MILD |  |  | NO | NA |  |
| 18 | c.494A>G | missense | MALE | SEVERE |  |  | NO | NA |  |
| 19 | c.499delC | frameshift | MALE | MODERATE |  |  | NO | NA |  |
| 20 | c.292-2A>T | splice site | MALE | MODERATE |  |  | NO | NA |  |
| 21 | c.3G>A | missense | FEMALE | SEVERE |  |  | NO | NA |  |
| 22 | c.558-3T>G | splice site | MALE | SEVERE |  |  | NO | NA |  |
| 23 | c.303C>A | nonsense | MALE | SEVERE |  | FTT | microcephaly | YES | Hypotonia |
| 24 | c.303C>A | nonsense | MALE | SEVERE |  | FTT | microcephaly | YES | Hypotonia |
| 25 | c.58G>T | nonsense | MALE | MILD | YES |  | NO | NO |  |
| 26 | c.27del | frameshift | MALE | SEVERE |  | FTT | NO | NA | Hypotonia |
| 27 | c.73-2A>G | splice site | MALE | MILD |  |  | NO | NO | Hypotonia |
| 28 | c.124C>T | nonsense | FEMALE | MILD |  |  | NO | NO |  |
| 29 | c.1A>G; p.Met1? | start loss | MALE | MILD |  |  | NO | NO | Hypotonia |
| 30 | c.94G>A | missense | MALE | MODERATE |  |  | NO | YES | Hypotonia |
| 31 | c.542del | frameshift | MALE | SEVERE | YES | Short Stature,Partial growth hormone deficiency,FTT | NO | YES | Hypotonia |
| 32 | c.101T>C | missense | FEMALE | MILD | YES |  | NO | YES |  |
| 33 | c.78_83dup | in-frame deletion | MALE | MILD |  |  | NO | NA |  |
| 34 | c.105T>A | missense | MALE | SEVERE |  |  | macrocephaly | YES | Hypotonia |
| 35 | c.409T>C | missense | MALE | MILD | YES |  | NO | YES |  |
| 36 | c.94G>A | missense | MALE | MODERATE |  |  | NO | YES |  |
| 37 | c.139C>T | nonsense | FEMALE | MILD | YES |  | NO | NA |  |
| 38 | c.558-2A>G | splice site | FEMALE | MILD |  | FTT | NO | NA |  |
| 39 | c.229G>A | missense | MALE | MILD |  | FTT | microcephaly | YES | Hypotonia |
| 40 | c.291G>A | missense | MALE | MILD |  |  | NO | YES |  |
| 41 | c.394_404del | frameshift | FEMALE | MILD |  | FTT,delayed bone age | macrocephaly | YES |  |
| 42 | c.2T>A;p.Met1? | start loss | FEMALE | MILD | YES |  | NO | NA | Hypotonia |
| 43 | c.181G>T | nonsense | MALE | MILD | YES | FTT | macrocephaly | NA | Hypotonia |
| 44 | c.256C>T | missense | FEMALE | NO |  |  | NO | NA |  |
| 45 | c.316T>G | missense | MALE | MILD |  |  | macrocephaly | YES | Hypotonia |
| 46 | c.557+1G>A | splice site | MALE | SEVERE |  |  | NO | NA |  |
| 47 | c.94G>A | missense | MALE | MODERATE | YES |  | NO | NA | Hypotonia |
| 48 | c.27G>A | nonsense | MALE | MILD |  |  | NO | NO |  |
| 49 | c.558-2A＞G | splice site | MALE | MILD |  |  | NO | NA |  |
| 50 | c.494A>G | missense | FEMALE | SEVERE |  |  | NO | YES | Hypotonia |
| 51 | c.94G>T | missense | MALE | MILD |  |  | microcephaly | YES | Hypotonia |
| 52 | c286C>T | nonsense | FEMALE | MILD |  |  | NO | NO |  |
| 53 | c.108dup | frameshift | MALE | MILD | YES |  | NO | YES | Hypotonia |
| 54 | c.494A>G | missense | MALE | SEVERE | YES | FTT,Growth Delay | NO | YES | Hypotonia |
| 55 | c.27G>A | nonsense | MALE | MILD |  |  | NO | NO |  |
| 56 | c.368-2A>G | splice site | FEMALE | MILD |  |  | NO | NO | Hypotonia |
| 57 | c.181_183del | missense | FEMALE | SEVERE |  |  | microcephaly | YES | hypertonia |
| 58 | c.332G>A | missense | FEMALE | SEVERE |  |  | microcephaly | YES | hypertonia |
| 59 | c.116T>G | missense | FEMALE | MILD |  | FTT,Growth Delay | NO | YES |  |
| 60 | c.384_394del | frameshift | FEMALE | NO |  | FTT,Growth Delay | microcephaly | YES |  |
| 61 | c.1A>G(p.Met1?) | start loss | MALE | SEVERE |  | Short Stature | NO | NO |  |
| 62 | c.100delT | frameshift | MALE | NO |  | Short Stature | NO | NO |  |
| 63 | c.332G>C | missense | MALE | MODERATE |  | Short Stature | NO | NO |  |
| 64 | c.332G>C | missense | MALE | MILD |  | Short Stature | NO | NO |  |
| 65 | c.158_159insA | frameshift | MALE | SEVERE |  | Short Stature | NO | NO |  |
| 66 | c.634_635del | frameshift(mosaic) | MALE | MILD |  |  | NO | NO |  |
| 67 | c.142C>T | nonsense | FEMALE | MILD |  |  | NO | NO |  |

**Table S1-2 The Genotype and Clinical Phenotype of Patients with CSNK2B Mutations**

| PATIENT NUMBER | EPILEPSY | AGE AT ONSET  (month) | SEIZURES | PROVOKING FACTORS | CLUSTER | EEG | MRI | drug-resistant/  multiple medications  （YES/NO） | ASMs |
| --- | --- | --- | --- | --- | --- | --- | --- | --- | --- |
| 1 | NO |  | NA |  |  | NO | NO |  | NA |
| 2 | YES | 18.00 | My, Fo seizure | Sonic stimulation | Yes | Slow background，generalized spike wave | White matter hyperintensity | YES | LTG, VPA, LEV,  CLB, ZNS |
| 3 | YES | early infancy | My, Fo seizure | Sonic stimulation | Yes | NO | NO | NO | VPA, LEV |
| 4 | YES | 2.00 | My, SPASMS(?), GTCS, TS,FBTCS | Photic and Sonic stimulation | Yes | Focal spike wave,generalized spike wave | Cerebellar atrophy | YES | CBZ,ZNS, CLB,VPA,ESM,NZP, PHT, PB, ACTH,TPM, LEV, LTG,  RUF, KD |
| 5 | YES | 0.10 | Fo seizure |  | Yes | Focal spike wave | Mega cisterna magna | YES | PB, CBZ, LEV,  TPM,VPA, LTG, KD |
| 6 | YES | 12.00 | My, GTCS | Fever |  | Generalized spike wave and poly spike wave | NO | YES | VPA,CZP, LEV, TPM |
| 7 | YES | 4.00 | GTCS | Fever | Yes | NO | Slightly widened subarachnoid space | NA | NO THERAPY |
| 8 | YES | 5.00 | GTCS |  | Yes | NO | Delayed myelination | NO | OXC, LEV |
| 9 | YES | 2.00 | GTCS |  | Yes | Focal spike wave | NO | NO | OXC, LEV |
| 10 | YES | 6.00 | GTCS |  | Yes | NO | NO | NO | LEV |
| 11 | YES | 6.00 | GTCS |  | Yes | Multifocal spike wave | NO | NO | VPA |
| 12 | YES | 5.00 | GTCS |  |  | NO | NO | YES | OXC, TPM, LEV |
| 13 | YES | 5.00 | GTCS | Fever |  | NO | NO | NO | LEV |
| 14 | YES | 3.50 | GTCS |  | Yes | NO | NO | NO | TPM |
| 15 | YES | 11.00 | Myotonic-atonic |  | Yes | Bifrontal spike wave | NO | YES | LTG, OXC, TPM,CZP, PREDNISOLONE |
| 16 | YES | 4.00 | GTCS |  |  | Slow background | NO | NO | VPA |
| 17 | YES | 5.00 | GTCS |  |  | NO | NO | NO | VPA |
| 18 | YES | 0.15 | My |  |  | More sharp waves in the cen tral, apical, and midline areas | NO | YES | PB、TPM、LEV |
| 19 | NO |  | NA |  |  | NO | NO |  |  |
| 20 | YES | 4.00 | GTCS |  |  | Sharp and slow waves in the central, parietal, occipital, middle, and posterior temporal regions | Bilateral frontotemporal epidural space slightly widened and a small amount of subdural efusion | YES | DAP、PB、OXC |
| 21 | YES | 3.00 | GTCS |  |  | More sharp waves in the  left area | Abnormal signal shadow in the right temporal, occipital parietal lobe,hippocampus,and splenium of the corpus callosum | NO | PB、LEV |
| 22 | YES | 3.00 | GTCS |  |  | Sharp wave, spike wave, and  slow wave in the frontal,  central, and temporal area | NO | YES | PB、LEV、TPM |
| 23 | YES | 6.00 | My, atonic |  |  | a brief generalized discharge arising from a mildly slow background;Interictal EEG during sleep at age 6 years (individual 1), showing abundant multifocal and generalized spikes and polyspikes | NA | YES | LTG、VPA、ZNS |
| 24 | YES | 10.00 | My, atonic |  |  | Interictal EEG during sleep at age 6 years showing frontally predominant generalized polyspikes;Ictal EEG generalized polyspike discharge;polyspike and wave at 2-3 Hz | NA | YES | LTG, VPA |
| 25 | YES | 12.00 | GTCS |  |  | NA | NA | NO | PTH |
| 26 | YES | 4.00 | My,RSE |  |  | Status epilepticus at age 12 years (individual 4), characterized electrographically by generalized polyspikes and polyspike and wave at ~2 Hz | NA | YES | VPA, CLN, RUF, ZNS, PHT, PB, TPM, LCM, KD, VNS |
| 27 | YES | 26.00 | GTCS\ESES |  |  | frontally predominant generalized polyspikes | NA | NO | NA |
| 28 | YES | 4.00 | Fo seizure |  |  | NA | NA | NO | PB,OXC |
| 29 | NO |  |  |  |  | NA | T2 hyperintensity and restricted diffusion of pontine central tegmental tracts |  |  |
| 30 | NO |  |  |  |  | NA | Mild to moderate diffuse abnormal signal in the cerebral white matter, low volume ventral pons |  |  |
| 31 | YES | 10.00 | GTCS,My,Atypical absenses,RSE |  |  | NA | NA | YES | NA |
| 32 | NO |  |  |  |  | NA | Periventricular gliosis |  |  |
| 33 | YES | 5.00 | Absences,GTCS |  |  | NA | Two possible germinolytic cysts | YES | ZNS、VPA、LEV |
| 34 | NO |  |  |  |  | NA | NA |  |  |
| 35 | YES | 6.00 | Fo seizure,GTCS |  |  | NA | NA | NO | VPA to 3 years |
| 36 | YES | 24.00 | Absences,tonic-spasms |  |  | NA | NA | YES | NA |
| 37 | YES | 24.00 | drop attacks, head drops with staring of eye |  |  | NA | NA | YES | EXT,CLB |
| 38 | YES | NA | Focal GTCS, drop attacks |  |  | NA | NA | YES | LEV、LTG |
| 39 | NO |  |  |  |  | NA | NA |  |  |
| 40 | YES | 4.00 | Fo seizure;GTCS |  |  | NA | NA | YES | NA |
| 41 | YES | 36.00 | GTCS |  |  | NA | NA | NO | NA |
| 42 | YES | 12.00 | unknown |  |  | NA | NA | YES | KD、LEV |
| 43 | YES | 2.00 | GTCS |  |  | NA | NA | YES | PHT、LTG、FBM |
| 44 | YES | 14.00 | Myoclonic-absences |  |  | NA | NA | NO | ZNS |
| 45 | NO | 37.00 | Febrile |  |  | NA | NA |  |  |
| 46 | YES | 2.50 | GTCS,My,Absenses,Tonic,RSE |  |  | NA | NA | YES | NA |
| 47 | YES | 84.00 | absences |  |  | NA | NA | YES | VPA、ETX |
| 48 | YES | 7.00 | GTCS,FS |  | Yes | Bifrontal spike wave,multifocal spike wave,generalized spike wave | NO | NO | VPA, CBZ |
| 49 | YES | 3.00 | GTCS |  | Yes | NO | NO | YES | LEV,VPA,OXC |
| 50 | YES | 0.50 | My |  |  | NA | NO | YES | NA |
| 51 | YES | 9.00 | Atonic |  |  | Disorganized base activity | NO | NA | NA |
| 52 | YES | 16.00 | GTCS |  | Yes | Ictal EEG highlighted a left temporo-posterior focus with subsequently generalized spike–wave discharges.The interictal EEG showed a left temporo-posterior focal discharge | NO | NO | VPA |
| 53 | YES | 20.00 | GTCS,Fo seizure |  |  | d multifocal spikes at onset and diffuse sharp waves at follow-up | NO | NO | VPA |
| 54 | YES | 0.23 | Fo seizure |  |  | The EEG at onset was abnormal with focal anomalies, mostly on the right side. EEG at follow-up showed paroxysmal multifocal activity | Hypoplasia of the cerebellar worm, delayed myelinization, and a mega cisterna magna | NO | LEV |
| 55 | YES | 7.00 | GTCS,Fo seizure |  | Yes | d multifocal spikes and generalized sharp | NO | NO | VPA |
| 56 | YES | 10.00 | GTCS |  |  | at onset was normal. The follow-up EEG showed some diffuse slow waves during sleep and drowsiness | NO | NO | VPA |
| 57 | NO | 8.00 | Febrile seizures |  |  | NA | Hypoplasia of the corpus callosum and pons, and enlargement of the cerebrospinal fluid spaces | NO | VPA |
| 58 | YES | 16.00 | GTCS,SPASMS |  |  | at onset showed focal and multifocal discharges with burst suppression | Gyral simplification and delayed myelination | NO | LEV |
| 59 | YES | 65.00 | absences |  |  | typical spike–wave complexes(3 Hz),at follow-up highlighted multifocal epileptiform discharges | Chiari type I malformation and syringomyelia. | NO | VPA,LTG |
| 60 | YES | 9.00 | Atonic |  |  | Diffuse anomalies and rare slow waves | NO | NO | VPA |
| 61 | YES | 3.00 | Fo seizure |  | Yes | Diffuse slow spike, multiple slow spike, and sharp wave discharges | NO | NO | LEV,TPM |
| 62 | YES | 5.00 | GTCS |  | Yes | Slow background and occasionally, an atypical spike | NO | NO | VPA,LEV |
| 63 | YES | 13.00 | My |  | Yes | Diffuse high-amplitude pike wave | Slightly widen of lateral ventricles | NO | LEV |
| 64 | NO |  |  |  |  | NA | NO |  | NA |
| 65 | YES | 2.00 | Fo seizure |  | Yes | Epileptiform discharge in the left anterior temporal and middle temporal regions | NO | NO | LEV |
| 66 | YES | 23.00 | Fo seizure,My,GTCS | Fever | Yes | Slow background, diffuse spike-slow, multiple spike-slow, and sharp wave discharges | Slightly widened transparent diaphragm | YES | LEV，OXC，TPM，VPA,LTG |
| 67 | YES | 17.00 | My |  | Yes | slow background, multifocal spikes, diffuse spike-slow, multiple spike-slow, and sharp wave discharges | Delayed myelination | NO | VPA |

ASM = anti-seizure medication; CLB = clobazam; CLN = clonazepam; DQ = developmental quotient; ETX = ethosuximide; FBM = felbamat; FTT = failure to thrive; GTCS= generalized tonic clonic seizure; ID/DD = intellectual disability/developmental delay; KD = ketogenic diet; LCM = lacosamide; LEV = levetiracetam; LTG = lamotrigine;NA=not acquired,NO=normal,MRI = magnetic resonance imaging; OXC = oxcarbazepine; PB = phenobarbital; PHT = phenytoin; RSE = refractory status epilepticus; RUF = rufinamide; TPM = topiramate; VNS = vagal nerve stimulator; VPA = valproic acid; ZNS = zonisamide

**Table S2. Cases and Literature**

| Case | Literature |
| --- | --- |
| 1-2 | CSNK2B splice site mutations in patients cause intellectual disability with or without myoclonic epilepsy |
| 3 | Truncatingmutation in CSNK2B andmyoclonic epilepsy |
| 4-5 | Identification of de novo CSNK2A1 and CSNK2B variants in cases of global developmental delay with seizures |
| 6-14 | Germline de novo variants in CSNK2B in chinese patients with epilepsy |
| 15 | Poirier–Bienvenu neurodevelopmental syndrome: A report of a patient with a pathogenic variant in CSNK2B with abnormal linear growth |
| 16 | Infantile Generalized Epilepsy Complicated with Psychomotor Retardation Induced by CSNK2B Gene Mutation: A Case Study and Literature Review |
| 17-22 | Clinical and genetic analysis of six Chinese children with Poirier‑Bienvenu neurodevelopmental syndrome caused by CSNK2B mutation |
| 23-47 | CSNK2B: A broad spectrum of neurodevelopmental disability and epilepsy severity |
| 48 | Developmental and epilepsy spectrum of Poirier–Bienvenu neurodevelopmental syndrome: Description of a new case study and review of the available literature |
| 49 | Epilepsy induced by CSNK2B gene combined with KCNQ2 gene mutation in an infant: a case report |
| 50-51 | Two different presentations of de novo variants of CSNK2B: two case reports |
| 52-60 | Expanding Phenotype of Poirier–Bienvenu Syndrome: New Evidence from an Italian Multicentrical Cohort of Patients |
| 61-65 | De Novo CSNK2B Mutations in Five Cases of Poirier–Bienvenu Neurodevelopmental Syndrome |
| 66-67 | Our cases |
| 15 | Baylor College of Medicine |
| 16 | Baylor College of Medicine |
| 17 | University of Pittsburgh Medical Center |
| 18 | New York University |
| 19 | Geisinger Medical Center |
| 20 | Mayo Clinic |
| 21 | Mayo Clinic |
| 22 | Translational Genomics Research Institute |
| 23 | University of Zurich |
| 24 | Boston Children's Hospital |
| 25 | University of Melbourne |
